# Anti-diarrhoeal activity of a polyherbal formulation in rats and elucidation of its cellular mechanisms 

**Published:** 2020

**Authors:** Sasikumar Murugan, Divya Purusothaman, Edwin Jothie Richard, Nehru Sai Suresh Chalichem, Bharathi Bethapudi, Prasanna Raja Chandrasekaran, Chandrasekaran Chinampudur Velusami, Prashanth D’Souza, Deepak Mundkinajeddu

**Affiliations:** 1 *Research and Development centre, Natural Remedies Pvt Ltd, Plot No. 5B, Veerasandra Indl. Area, 19th K.M. Stone, Hosur Road, Bangalore 560100, Karnataka, India*

**Keywords:** Anti-diarrhoea, Anti-secretory, Castor oil, Camp, Diarrhoeal drop, Enterotoxin, Stodi

## Abstract

**Objective:**

The present study was aimed to study anti-diarrhoeal activity of a polyherbal formulation (PHF) in rats and elucidate its mechanism of action.

**Materials and Methods:**

Anti-diarrhoeal activity of PHF was investigated using castor oil-induced diarrhoea, small intestinal transit and enteropooling models in rats. PHF was tested at 75, 150 and 300 mg/kg rat body weight. Loperamide was used as a reference control for *in vivo* studies. Anti-secretory action was evaluated against heat labile enterotoxin (from *Escherichia coli*) induced secretion in rat ileal loop model. The effect of PHF (12.5-100 µg/ml) on cAMP-dependent secretory activity was investigated against forskolin-induced cAMP release in HT-29 cells.

**Results:**

PHF demonstrated significant (p≤0.05) anti-diarrhoeal activity by increasing the time for first faecal drop and inhibited diarrhoeal episodes by 43, 58 and 60% at 75, 150 and 300 mg/kg body weight, respectively in a dose-dependent manner. Also, the intestinal transit was inhibited upto 33% and the weight of secretory contents induced by castor oil was significantly reduced by PHF, approximately 29% in enteropooling assay. On the other hand, the intestinal loop instilled with PHF and enterotoxin from *E. coli *demonstrated 61% inhibition of fluid accumulation as compared to loop instilled with enterotoxin only. *In vitro *studies indicated that PHF inhibits cAMP release in HT-29 cells corroborating the anti-secretory effects observed in aforesaid studies.

**Conclusion:**

The results suggest that the PHF possesses anti-diarrhoeal activity, evident through reduced faecal output, decreased intestinal transit and anti-secretory activities.

## Introduction

Diarrhoea, the word seems to be originated from Greek and Latin languages (Greek: *dia*, through Latin: *rheein*, to flow or run), involves the passage of loose or watery stools. Increased frequency of bowel is its characteristic feature with/without the presence of blood in stools. Diarrhoea occurs as a result of compact living conditions mingled with less prioritised hygiene and malnutrition in developing countries (Walker et al., 2011 ▶). The plausible contributing factors for diarrhoea are diversified and can vary from pathogen to immunological and nutrition-related factors but commonly, it occurs due to various pathogens like bacteria, virus and parasites which can disturb homeostatic environment of gastro intestinal tract by entering through food, water and unhygienic environment (Sharma et al., 2012 ▶; Whyte and Jenkins, 2012 ▶). 

Widely and commonly, diarrhoea is classified as osmotic/secretory type or diarrhoea secondary to altered intestinal motility (Woods, 1990 ▶). Osmotic diarrhoea is characterised by passive movement of more fluids into the intestinal lumen due to osmotic gradient created by the osmotically active substances in the intestinal lumen. Whereas, in secretory diarrhoea the fluid content of lumen is due to the activation of secretory pathways by pathogens or by the native malfunctionalities of the gut enterocytes (Whyte and Jenkins, 2012 ▶). 

The use of alternative approaches remain as a good option to create a solution for the limitations posed by the conventional (allopathic) anti-diarrhoeal therapeutic agents. For example, racecadotril and loperamide are in use to treat secretary diarrhoea but they have the limitations like bronchospasm, vomiting, fever and increase the content of infectious agent in the jejunum (Fischbach et al., 2016 ▶). Different antibiotics are currently in use to fight this condition; however, drug resistance/disturbance in normal flora is another issue to think about (Rafii et al., 2008 ▶; Qu et al., 2016 ▶). Thus, it clearly emphasises the need for safe anti-secretory and anti-motility product and the natural products can provide safe alternative for synthetic compounds.

Infusions, decoctions and enemas prepared by using different parts of various herbs, can be considered under alternative and traditional treatment strategy. Although the factor of unwanted effects cannot be eliminated completely from the therapeutic concept in the usage of herbs, still their consideration is a good choice which may be due to possibly low cost, easy access and availability, tolerance, multiple mode of actions and considerable safety profile. May be the lack of proper scientific validation makes the herbal approach strategy not convincing. Hence, the current research work involves testing the formulation made with herbs by preserving their holistic nature, as an alternative scientific strategy for diarrhoeal condition.

The present study was aimed to investigate the possible anti-diarrhoeal activity of polyherbal formulation (PHF) in various rat models *viz*., castor oil- induced diarrhoea, charcoal intestinal transit and enteropooling tests in rat. Also, the mechanism of actions of PHF was investigated in rat ileal loop assay, *in vitro* chicken ileal contractility and forskolin induced cAMP release assay in HT-29 cells.

## Materials and Methods

**Plant material**

The investigational polyherbal formulation containing *Punica granatum*, *Acacia arabica*, *Andrographis paniculata*, *Holarrhena antidysenterica* and *Terminalia bellerica* is known as Stodi (mentioned as PHF), manufactured by M/S. Natural Remedies Pvt. Ltd., Bengaluru, India. The dried parts of the aforesaid plants were dried, grounded and blended homogenously in appropriate quantities and blend was subjected to gas chromatography for the quantification of marker compounds i.e., polyphenols and tannins. 

**Chemicals and reagents**

Castor oil (Srinivasa Enterprises, Bengaluru, India; B.No: 06); Carboxy methyl cellulose, Acetylcholine chloride (HiMedia Laboratories Pvt. Ltd., Mumbai, India); Loperamide hydrochloride capsules (Micro Labs ltd., India; Trade name: Eldoper^®^; Batch No.: ELAS0060).

 HT-29 cells were procured from American Type Culture Collection (Manassas, USA) and maintained as cryopreserved stocks in liquid nitrogen containers (-196°C); ELISA kit (GenScript, NJ, USA); DMSO (Ranchem, India), MTT (Sigma, India), Forskolin (Sigma, India), McCoy’s 5A (Thermofisher scientific, US) were procured. Sodium dihydrogen phosphate anhydrase, calcium chloride, Sodium chloride, sodium bicarbonate, dextrose anhydrous, potassium chloride and magnesium chloride were procured from HiMedia Laboratories Pvt. Ltd., Mumbai, India. Automatic organ bath (PANLAB, Spain) and Chart 5 software (V5.5.6 AD instruments, New Zealand) were used for measuring ileal contractions.

**Experimental animals**

Albino Wistar rats of either sex, aged 6-8 weeks inbred at Central animal facility, Natural Remedies Pvt. Ltd were kept for acclimatisation for a period of 5 days, prior to experimentation. Rats were maintained at room temperature of 22°C (3°C) and relative humidity between 30 and 70%. Twelve hours day and night cycle was maintained with automatic light system. Except during protocol defined periods, animals were allowed to have free access of UV purified water and rodent pellet feed *ad libitum.* All the experimental procedures were executed as per CPCSEA guidelines and approved by ethical committee (Approval number: IAEC/NR-PCL-03/07/10/17 and IAEC/NR-PCL-04/12/18).

**Castor oil-induced diarrhoea** ***in vivo***

Thirty rats were allocated randomly, to five groups (n=6 in each group). Group I was administered CMC (0.5%) at the rate of 10 ml/kg rat body weight. Group II was administered loperamide (5 mg/kg) as a single dose on day 5. Group III, IV and V were administered PHF at 75, 150 and 300 mg/kg respectively for five consecutive days. On day 5, following overnight fasting, castor oil was administered to all the rats at 1 ml/rat (orally), one hour after treatment. Rats were placed in individual cages lined with adsorbent paper and observed for time taken for first diarrhoeal drop, total number and weight of diarrhoeal faeces for 4 hrs (Tadesse et al., 2014 ▶; Jalilzadeh-Amin and Mahama, 2013 ▶). 

The Percentage inhibition of defaecation (%) was calculated using the formula:

=[A-B/A]×100

- Mean number of diarrhoeal faeces caused by castor oil administration

B- Mean number of diarrhoeal faeces observed after treatment

**Small intestinal transit in castor oil induced diarrhoea** ***in vivo***

Rats were allocated to six groups with six animals in each group. Group I and II were administered CMC (0.5%), while groups III, IV, V & VI were administered loperamide (5 mg/kg), PHF at 75, 150 and 300 mg/kg rat body weight respectively as a single dose. After 30 mins., of the treatment, overnight fasted rats were administered (except Group I) castor oil (1 ml per rat) orally. Thirty minutes after castor oil administration, charcoal meal (1 ml of 10% w/v in 0.5% CMC) was administered orally. After 20 minutes following charcoal meal, animals were sacrificed and distance travelled by charcoal meal in small intestine was measured (Agbor et al., 2014 ▶; Degu et al., 2016 ▶). The mean distance travelled for 100 cm length of small intestine is calculated and represented as peristalsis index.

The percentage inhibition of small intestinal transit was calculated using the formula:

=[A-B/A]×100

- Distance (Mean) travelled by charcoal meal in castor oil administered rats
- Distance (Mean) travelled by charcoal meal after treatment

**Enteropooling in castor oil-induced diarrhoea** ***in vivo***

Small intestine luminal fluid accumulation was measured by using Robert et al. (1976) ▶ protocol. Grouping was done by random allocation with six rats in each group. Overnight fasted rats were administered single dose of either loperamide (5 mg/kg) or PHF at 75, 150 and 300 mg/kg rat body weight respectively. After 1 hr, rats were administered with castor oil (1 ml/rat) and were sacrificed 30 min. post castor oil administration. Abdomen was incised and small intestine was excised with ligatures at pylorus (anterior) and ileo-caecal (posterior) junctions. The isolated intestine was weighed and the volume and weight of the intestinal contents were measured by using a graduated glass tube (Robert et al., 1976 ▶; Rahman et al., 2015 ▶).

The percentage inhibition of intestinal secretion was calculated using the formula:

=[A-B/A]×100

- Mean weight of the intestinal content in castor oil administered rats
- Mean weight of the intestinal content after treatment

**Ileal loop assay in enterotoxin induced diarrhoea** ***in vivo***

Albino Wistar rats (N=9) were allocated randomly to three groups, consisting of three animals per group. Group I was instilled with normal saline, Group II was instilled with heat labile enterotoxin from *E. coli* (10 µg) and Group III was instilled with enterotoxin and PHF (5 mg/ml at the volume of 0.5ml per loop). Closed loop rat model was used to evaluate the efficacy of PHF in inhibiting *Escherichia coli *enterotoxin induced intestinal fluid secretion. Briefly overnight fasted rats were anesthetised with cocktail mixture of ketamine and xylazine (35 mg/kg and 5 mg/kg; i.p.). A small abdominal incision was made and the intestine was exposed. Two ligatures were placed at 4-5 cm distances in the distal ileum to make closed loop in each rat. Following instillation of either saline or enterotoxin or enterotoxin+PHF in the respective groups, ileum was pushed back immediately into the abdominal cavity and sutured. Following recovery after 15 hrs, anesthetised rats were dissected and loop was excised, length and weight were recorded (Galindo et al., 2007 ▶; Lange, 1982 ▶).

***In vitro*** **Studies**

**Figure 1:**
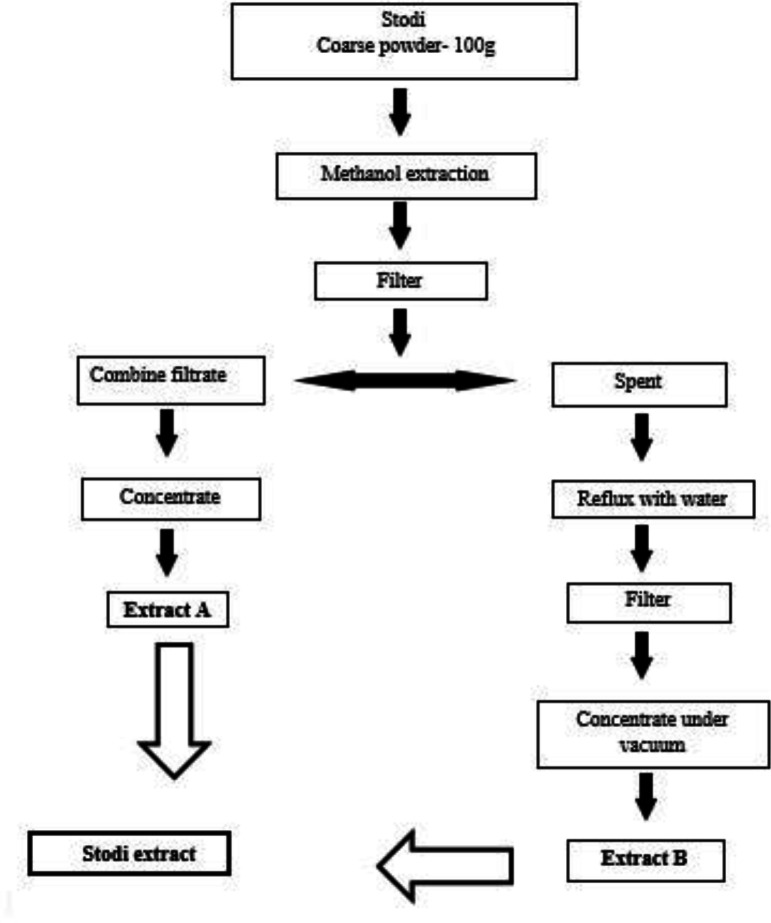
Schematic representation-extraction procedure of PHF for cAMP inhibition assay against forsokolin induction in HT29 cell

The extract of the formulation was prepared as mentioned in Figure 1, and was used for *in vitro* studies. Briefly coarse powder was subjected for extraction and subsequent reflux procedures. Finally extract A and B were mixed to prepare the final extract.

**cAMP inhibition assay** ***in vitro***

Cytotoxic assay: PHF was checked for cytotoxicity through 3-(4, 5-dimethylthiazol-2-yl)-2, 5-diphenyltetrazolium bromide (MTT) assay, up to a maximum soluble and non-precipitating concentration in HT-29 cells. For the assay, 96 well plate was seeded with 100,000 cells of HT-29 and kept for 24 hrs of incubation. Subsequently cells were subjected for treatment with extract that was prepared in serial dilution manner (12.5-100 µg/ml). After incubation, 10 µl of yellow MTT solution was added and allowed for incubation for about 1 hr and then absorbance was taken at 570 nm.

Percentage viability was calculated using formula:

$=(\mathrm{Test}\frac{\mathrm{OD}}{\mathrm{Control}}\mathrm{OD})\times 100$

The non-cytotoxic concentration of PHF (12.5-100 µg/ml) were tested in cAMP inhibition assay. In brief, the procedure followed was, human colorectal adenocarcinoma (HT-29) cells were adjusted to be 100,000 cells/well in McCoy's 5A supplemented with 10% FBS, and 500 μl of the cell suspension were seeded into 48-well culture plate and allowed for incubation at 37°C in a humidified 5% CO_2_ incubator for 24 hrs. Following incubation, the cells were treated with serial dilutions of the extract–(triplicate for each concentration) for 30 min and then incubated for further 1 hr with forskolin (1 µM). Following incubation, the cell supernatant was aspirated and removed and then washed twice with DPBS and incubated with 100 µl of cell lysis buffer. Cell lysate was subjected for centrifugation at 1000Xg for 10 min. and the supernatant was removed and used for the estimation of cAMP using ELISA technique (Schulzke et al., 2011 ▶). 

**Ileal contractions** ***in vitro***

Chicken ileum was collected from a slaughter house under the supervision of a veterinarian. Immediately after collection, the segments of ileum were flushed with physiological saline to remove the contents and extra tissue. The ileal segment was cut into small segments of 1cm length (approx.) and were placed in automatic multichannel organ bath filled with tyrode’s solution (Composition g/L: CaCl_2_-0.2, D-Glucose-1, MgCl_2_-0.1, NaCl-8, KCl-0.2, NaH_2_PO_4_-0.05 g, NaHCO_3_-1,) that was aerated with carbogen and maintained at 37°C. Ileal contractions were recorded isometrically using a force displacement transducer connected to polygraph (chart 5 software). The ileal segments were allowed to stabilise for 60 min. and maintained at under an optimal tension of 1g before experimentation. After the equilibration, ileum was exposed to acetylcholine chloride (300 nM-EC_50_ dose obtained from standardisation study) and contractile responses were recorded. This is considered as baseline contractile response induced by ACh. Subsequently tissue was washed and extract of PHF at 10, 30, 100, 300 and 1000 µg/ml was tested against acetylcholine (300 µg/ml) The percent inhibition of contraction induced by ACh was calculated (Borrelli et al., 2006 ▶; Sadraei et al., 2015 ▶).

**Data analysis**

The results were obtained through one way ANOVA followed by Bonferroni as post–hoc test by using SPSS and were expressed as mean±SD. In case of heterogeneous data, after transformation Dunnett T3 method was used. Statistical significance was set at p*≤*0.05.

## Results

**Effects of PHF on castor oil induced diarrhoea**

The mean time for onset of diarrhoea in rats administered with castor oil was observed as thirty-six minutes. Loperamide completely inhibited diarrhoea when compared to castor oil administered rats. While at 150 and 300 mg/kg doses of PHF, the onset of diarrhoea was significantly delayed, when compared with negative control (castor oil administered rats) (Table 1).

The mean number and weight of diarrhoeal faeces was nil in loperamide treated rats while PHF significantly exhibited dose dependent decrease in number and weight of diarrhoeal faeces as compared to rats treated with castor oil only. PHF exhibited 43, 58, and 60% inhibition of diarrhoea at 75, 150 and 300 mg/kg respectively as compared to castor oil treated rats (Table 1).

**Effect of PHF on small intestinal transit**

Distance travelled by charcoal was significantly increased in negative control group than normal control group. Loperamide significantly reduced the distance by 45.69%. While PHF at 150 and 300 mg/kg markedly reduced the distance travelled by charcoal to 31.53% and 38.66% respectively, when compared to castor oil administered rats (Table 2).

**Table 1:** Effect of PHF on castor oil induced diarrhoea in rat

**Group**	**Onset time of** ** diarrhoea (mins.)**	**Number of ** **diarrhoeal faeces**	**Weight of diarrhoeal** ** faeces (g)**	**Percentage inhibition of defaecation**
Diarrhoea control	36.00±4.14	8.83±0.75	11.17±1.80	NA
Loperamide hydrochloride (5 mg/kg, *p.o*.)	NA	0.00±0.00#	NA	100.00
PHF (75 mg/kg, *p.o*.)	47.83±5.45	5.00±1.26#	7.37±0.67#	43.40
PHF (150 mg/kg, *p.o.*)	59.17±14.24#	3.67±1.03#	5.58±0.96#	58.49
PHF (300 mg/kg, *p.o.*)	69.17±13.30#	3.50±1.37#	4.52±1.92#	60.38

NA–Not applicable. Data was represented as mean±SD. #denotes the significance difference at p*≤*0.05

**Table 2:** Effect of PHF on small intestinal transit

**Group**	**peristalsis index (%)**	**%Inhibition**
Normal control	56.77±4.50	NA
Diarrhoea control	83.95±11.50*	NA
Loperamide hydrochloride (5 mg/kg, *p.o.*)	45.59±5.63#	45.69
PHF (75 mg/kg, *p.o.*)	71.51±14.50	14.82
PHF (150 mg/kg, *p.o.*)	57.48±18.32#	31.53
PHF (300 mg/kg, *p.o.*)	51.50±10.55#	38.66

NA–Not applicable

Values were expressed as mean±SD (n=5-6). Analysis was performed by One-Way ANOVA using SPSS. Loperamide group and Test doses (75, 150 and 300 mg/kg) were compared to diarrhoea control group to denote the treatment efficacy (^#^p≤0.05)and diarrhoea control group was compared to normal control group to check the intensity of induction (^*^p≤0.05).

**Effect on castor oil-induced enteropooling**

In the gastrointestinal enteropooling test, weight and volume of intestinal content significantly increased in castor oil administered rats in comparison to normal control group. Loperamide significantly decreased the volume and weight of intestinal content as compared to diarrhoeal control. While PHF treatment significantly reduced the weight of the intestinal content and where as non-significant reduction was observed in intestinal volume as compared to diarrhoeal control. The percentage inhibition of diarrhoea was found to be 54.68, 21.71, 29.81 and 28.54 for loperamide and PHF at 75, 150 and 300 mg/kg respectively, compared to diarrhoeal control group (Table 3).

**Ameliorating effect of PHF on entero-toxin induced fluid accumulation**

The heat labile enterotoxin from *E. coli* instilled into rat ileal loop, increased the net fluid accumulated and was considered 100% with respect to the fluid accumulated in untreated rats. The ileal loop instilled with PHF and heat labile *E. coli *significantly inhibited the net fluid accumulation by 82% and the net weight of the ileal loop contents by 61% (Table 4).

**Effect of PHF on cAMP levels** ***in vitro***

PHF did not show any toxicity at the tested concentrations (12.5-100 µg/ml) to HT-29 cells (data not shown). PHF exhibited significant inhibitory effect (23%) on forskolin induced cAMP release in HT-29 cells (Table 5).

**Effect of PHF on ileum contractions** ***in- vitro***

The contractile response of ileum to acetylcholine (300 nM) was recorded. The amplitude of contraction reduced with PHF at 300 and 1000 µg/ml in the presence of ACh were recorded. The force of contraction induced by ACh was reduced by PHF to 23 and 27% at 300 and 1000 µg/ml respectively (Table 6).

**Table 3:** Effect of PHF on fluid accumulation in enteropooling

**Treatment groups**	**Volume of intestinal fluid (ml)**	**Weight of intestinal content (g)**	**Percentage inhibition of diarrhoea (%)**
Normal control	1.08±0.17	1.27±0.31	NA
Diarrhoea control	3.18±0.56*	3.16±0.53*	NA
Loperamide hydrochloride (5 mg/kg, *p.o.*)	1.40±0.34#	1.43±0.15#	54.68
PHF (75 mg/kg, *p.o.*)	2.58±0.28	2.47±0.26	21.71
PHF (150 mg/kg, *p.o.*)	2.32±0.77	2.22±0.71#	29.81
PHF (300 mg/kg, *p.o.*)	2.22±0.37	2.26±0.40#	28.54

NA–Not applicable

Values were expressed as mean±SD (n=5-6). Analysis was performed by One-Way ANOVA followed by Dunnett T3. All the diarrhoeal control group parameters were compared to normal control (*p≤0.05) and treatment groups were compared to diarrhoeal control group (#p≤0.05).

**Table 4:** Effect of PHF on fluid accumulation in enterotoxin induced diarrhoea

**Treatment groups**	**Weight (g/cm)**		**Volume (ml)**	
	**%Control (Mean±SD)**	**%Inhibition**	**%Control (Mean±SD)**	**%Inhibition**
Normal control	---	---	---	---
Enterotoxin (10 µg)	100.0±1.4	---	100±22	---
PHF (5 mg/ml)	39.0±4.0	61	18±7.9	82

**Table 5:** Effect of PHF on cAMP inhibition

**Test article**		**Concentration (µg/ml)**	**cAMP (pM) Mean±SD**	**%inhibition**
PHF	+ Forskolin (1 µM)	12.5	77.4±2.81*	23
		25	76.8±7.13*	23
		50	77.8±2.94*	22
		100	80.2±3.19*	20
DMSO		0.1%	100.0±2.72	---

*p<0.05 vs DMSO

**Table 6:** Effect of PHF on ileal contractions

**Test substance**	**Ileal contractile response (g)**				**Percentage relaxation (%)**
	**Tissue 1**	**Tissue 2**	**Tissue 3**	**Average**	
Acetylcholine chloride (300 nM)	2.11	2.92	3.28	2.77	-
PHF (10 µg/ml)	2.13	2.95	3.25	2.78	-
PHF (30 µg/ml)	1.98	2.81	3.48	2.76	-
PHF (100 µg/ml)	1.91	2.75	3.60	2.75	-
PHF (300 µg/ml)	1.39	2.13	2.65	2.06	22.7
Acetylcholine chloride (300 nM)	1.82	2.50	3.67	2.66	-
PHF (1000 µg/ml)	1.12	1.87	1.88	1.62	26.5
Acetylcholine chloride (300 nM)	1.68	2.35	2.61	2.21	-

## Discussion

Diarrhoea may be an acute or chronic condition characterised by frequent passage of liquid faeces in association with abdominal cramps and loss of electrolytes due to perturbed homeostatic condition between absorptive and secretory mechanisms of intestinal tract. The current research work was aimed to validate scientifically, anti-diarrhoeal properties of PHF and to elucidate its mechanism of actions through *in vitro *and *in vivo* systems.

The study findings indicate anti-diarrhoeal activity of PHF evidenced from 60% of inhibition of defecation. In addition the other studies like anti-secretory and anti-motility actions were also proved.

Current therapeutic regimen mainly involves synthetic anti-motility drugs or antibiotics etc., to treat various types of diarrhoeal conditions. Although they provide immediate comfort from pathological condition, it is not devoid of adverse effects as they can cause disturbance in normal flora of GIT that are essential for healthy growth and wellbeing (Faure, 2013 ▶; Fischbach et al., 2016 ▶). However, formulations of plant origin are in use since ancient times without noticeable remarks and moreover prestigious organisations like WHO is also encouraging use of herbal formulations/plant derived formulations as solutions for global health (WHO, 2019 ▶).

Castor-oil induced diarrhoea in rat model was implemented for current research work to study anti-diarrhoeal effects of PHF and a series of different studies were also executed to understand possible mechanism of actions of PHF. Usage of castor oil was well documented as an inducing agent to study various anti-diarrhoeal actions of test substances (Akter et al., 2013 ▶; Tadesse et al., 2014 ▶) due to its multi-dimensional nature in promoting the pathological condition. Castor oil is a vegetable oil whose constituent called as ricinoleic acid, produced as a metabolite by the action of lipases, has been proved as a responsible factor for its diarrhoea inducing capability. Ricinoleic acid induces hypersecretory response, by mechanisms like activation of adenylate cyclate or active secretion mediated by cAMP of the mucosal membrane, stimulation of PGs (prostaglandins) (Hardman and Limbird, 2001 ▶; Tunaru et al., 2012 ▶; Capasso et al., 1992 ▶; Uchida et al., 2000 ▶). In consistent with previous results, administered dose of castor oil significantly induced the diarrhoea in the current research work also (Meite et al., 2009 ▶). 

In the current study, anti-diarhoeal effects of PHF were tested in castor oil induced diarrhoea model in which it showed 60.38% percentage inhibition of defaecation along with decreasing the wet faeces significantly. This decrease in wet faeces was a reflect for anti-secretory action of PHF. Similarly, kunal et al. reported that 42% inhibition of defecation by kutaja parpati vati (500 mg/kg dose) in rats (Kunal et al., 2012). In another research study by Degu et al. revealed that chloroform and methanol fractions of *Croton macrostachyus* at 200 and 100 mg/kg, showed 70% and 46% inhibition of defecation respectively (Degu et al., 2016 ▶). The research work by Mekonnen et al. emphasised the methanolic extract of *Justicia schimperiana, *as an anti-diarrhoeal agent in castor oil model, as it showed the percentage inhibition of 39% and 51% at 100 and 200 mg/kg doses respectively (Mekonnen et al., 2018 ▶). 

The findings from the anti-diarrhoeal studies reported elsewhere indicate that herbs/herbal extracts have anti-diarrhoeal activity. The percentage inhibition ranges from 40-70%, where higher activity was observed for extracts. While in the current study, the test substance was a PHF, which is a holistic formulation not an extract but could show efficacy of 60% inhibition.

Since it was evidenced that most of the diarrhoeal cases are infectious type that are characterised by excessive fluid accumulation due to enhanced secretion in the lumen, the current study investigated the efficacy of PHF in ameliorating the fluid accumulation induced by pathogen or its toxin. The virulence of enterotoxigenic *Escherichia coli* that is responsible for infectious diarrhoea, is due to its toxin which is a heat labile. Structurally, the toxin has two types of sub units, A (single) and identical B-sub units (five). It induces the pathology by binding to ganglioside GM1 [Galβ1-3 GalNAcβ1-4 (Neu5Acα2-3) Gal-β1-4Glc-Ceramide], of the intestinal epithelial cells through its B-subunit which leads to the translocation of A-sub unit, results in the elevation of cAMP through cascade of events (Minke et al., 1999 ▶). This elevated level of cAMP attributes to the hyper secretive state of the intestine. It was very clearly elucidated in the current study that PHF decreased enterotoxin induced fluid accumulation in the lumen, hence has the ability to inhibit secretory diarrhoea. This might be due to tannins of *Punica granatum*, since tannins promote the formation of protein tannate (denatured protein) that in turn reduces mucosal secretion (Kavitha et al., 2003 ▶). The weight of intestinal loop is considered to be a more accurate indicator of secretory action as the weight of the loop will take into consideration the intercellular fluid accumulated in the intestinal wall (Lange, 1982 ▶). To further reinforce the exact pharmacodynamic nature of PHF on this aspect, it has been carried forwarded to see the effect of test substance on elevated cAMP levels. Results from *in vitro* cAMP study reveal that PHF has the ability in downregulating the cAMP levels induced by forskolin. 

Deconjugation of bile acids due to gluten or ill health also elevates cAMP results in chronic watery diarrhoea (Fan and Sellin, 2009 ▶). Since PHF has been proved for its nature against cAMP, it can be a choice of candidate to treat chronic conditions. Chen et al., also emphasized the importance of herbs in ameliorating toxin induced diarrhoea. In their study, they concluded that ginger and its constituents were effective against enterotoxin induced diarrhoea but they act by interrupting the mucosal binding of toxin (Chen et al., 2007 ▶). In another study, researchers have claimed the anti-diarrhoeal action of *Holarrhena antidysenterica *seed extract was comparable to gentamicin against *E. coli *induced diarrhoea (Sharma et al., 2015 ▶). Thus, herbs were proven to have anti-diarrhoeal actions by inhibiting secretion. PHF also demonstrated anti-diarrhoeal effects via anti-secretory mechanism plausibly by down regulating cAMP. 

Homeostatic cross link between secretion and absorption is maintained by various hormonal mechanisms and normal epithelial function which are disrupted by stress and anxiety that alters intestinal motility, finally results in altering the absorption leading to diarrhoea (Vasina et al., 2006 ▶; Sheikh et al., 2018 ▶). As described earlier ricinoleic acid of the castor oil enhances smooth muscle contraction/motility. The peristaltic action induced by castor oil was successfully inhibited by the PHF at all the tested doses. The peristaltic inhibitory action shown by highest dose of PHF is almost nearer to loperamide. The anti-motility effects are evident from the effects of PHF observed in charcoal meal test. In addition, *in vitro* studies on chick ileum revealed that PHF inhibited muscle contractions induced by acetylcholine.

PHF revealed significant anti-diarrhoeal activity in rodent model, plausibly by anti-secretory and anti-motility activities. This dual mechanism could have been possible due to multi-pronged target approach exhibited by polyherbal formulation.

## Conflicts of interest

All the rights are reserved by Natural Remedies Pvt. Ltd, as authors are employed by the organisation.
